# Body Measurements and Body Condition Scoring in Bactrian Camels

**DOI:** 10.3390/ani15213157

**Published:** 2025-10-30

**Authors:** Bernard Faye, Shynar Akhmetsadykova, Moldir Akhmetzhanova, Zauresh Bilal, Zhaidar Musayev, Gaukhar Konuspayeva

**Affiliations:** 1 Center of International Cooperation on Agriculture Research for Development—CIRAD, UMR SELMET, Campus International de Baillarguet, 34398 Montpellier, France; 2 LLP “Scientific and Production Enterprise Antigen”, Almaty 040905, Kazakhstan; shynar.akhmetsadykova@gmail.com (S.A.); a.moldir.88@mail.ru (M.A.); bilalzauresh@gmail.com (Z.B.); antigen.chem@gmail.com (Z.M.); konuspayevags@hotmail.fr (G.K.); 3 LLP “Kazakh Research Institute for Livestock and Fodder Production”, Almaty 050035, Kazakhstan; 4 Department of Biotechnology, Al-Farabi University, Almaty 050040, Kazakhstan

**Keywords:** Bactrian camel, hump, body condition assessment, body measurements, feeding management, animal welfare

## Abstract

**Simple Summary:**

Body measurements and body condition score are common tools used by different stakeholders to assess the phenotypes and the global conditions of farm animals. While few references to body measurements were published, the scoring of body condition was not available for the Bactrian camel. After giving some information on the effect of age on body measurements, the present paper proposes a grid to evaluate the status of the animal by using a 6-point scoring system, from very emaciated Bactrian camels to very fat ones. This scoring is based on the observation of 12 anatomical points from the side and from behind. It will be necessary to validate the reproducibility and repeatability of the method.

**Abstract:**

Body measurements are used regularly to describe phenotypes or the weight of animals. However, little data describe the age effect on the different measurements. In the present paper, 477 Bactrian camels (7–180 months old) were measured (length of the head, length and circumference of the neck, height at the withers, girth circumference, length of the front leg, and length of the body), and their growth was modeled by comparing different equations, the most convenient being the beta growth equation. In addition, the differences in body measurements of adult Bactrian camels between farms were tested by variance analysis, and multivariate analyses were used for identifying homogeneous clusters of camels according to their body measurements. The sampled animals were from different regions of the country. The significant relationships between cluster and region support the idea of racial differentiation. Body condition scoring is widely used in farm animals as an indicator of their feeding, health, and welfare status. If a body condition score is available for dromedaries, there is no specific grid for the Bactrian camel. Starting from typical examples taken from photos in different conditions, including extreme ones, a representation of a scoring system of 6 points from very emaciated (score 0) to overweight Bactrian camel (score 5) was proposed in the form of drawings from the right side, back, and three-quarter rear. To complete the drawings, a descriptive grid was built as a base for training stakeholders in relation to Bactrian camel farm management. However, the seasonal change in the fleece abundance, the variability of the humps’ shape, and the presence of crossbreeding with dromedary camels require adapting the scoring accordingly.

## 1. Introduction

Body measurements (BM) are generally used to differentiate phenotypes among domesticated animals. In camelids, phenotype description based on body measurements was widely proposed in the dromedary camel [1,2,3,4,5], but more rarely in the Bactrian camel [6,7,8], and generally on a limited number of animals. Yet, BM are a basic method to describe the biodiversity within a species, and are a classical means to achieve breed description. Moreover, BM can be a useful tool to estimate the weight of the animals in field conditions where the use of weighing material for heavy animals, such as camels, is difficult to manage. If several equations were suggested to predict the weight of the dromedary camel from specific body measurements [9], to our knowledge, only one reference used BM to estimate the weight of the Bactrian camel [10].

The body condition score (BCS) is a low cost-effective method used by scientists and technical advisors worldwide to assess the nutritional status of farm animals. Indeed, it is considered that body condition can reflect the importance of fat reserves and, indirectly, the nutritional balance of the diet, health status, and reproductive capacity. The interest of BCS was emphasized for assessing the animal’s conditions during the important physiological steps of their life cycle: breeding, parturition, beginning of lactation, and physical activities (agricultural works, competition, etc.). It is also useful as an indicator of the availability and quality of natural resources, to assess their adequacy with the requirements of the animal, especially in extensive pastoral systems during drought [11]. The modern alternative methods, such as imaging techniques or ultrasonography, are not necessarily easy to use in pastoral conditions and are most costly [12].

The scoring grid was set up for a long time for European *Bos taurus* [13], or tropical *Bos indicus* [14], and more recently for tropical *Bos taurus* [15]. It was also proposed for other farm animals such as sheep [16], goat [17], horse [18], donkey [19], and even buffalo [20]. A recent review also proposed a standardized scoring system in tropical farm animals [21], notably because the number of scoring levels, the visual anatomical zones to be assessed, or the additional investigations, such as palpation, were heterogenous. Regarding the camelid family, BCS was applied in alpacas and llamas [22].

Regarding camel, the only BCS in dromedary (*Camelus dromedarius*) was published [23,24] and used in different publications in Australia [25] and in Spain [26]. To our knowledge, there was no equivalent for the Bactrian camel (*Camelus bactrianus*). Compared to the other farm animals, the large camelids have a specificity, leading to the impossibility of applying the classical grid used for bovine: the presence of one (dromedary) or two humps (Bactrian), where are concentrated the main part of the fat reserves [27]. Moreover, the behavior of the hump in case of important weight loss is different according to species: the hump of the dromedary is melting while the humps of the Bactrian are “falling”. In addition, the only slight appreciation of fat condition in the Bactrian camel, which was published, was based on the status of the hump observed backward [28]. In this reference, only 4 levels were described: (i) very fat camel with well-raised hump, (ii) medium fat camel with hump slightly leaning on the side, (iii) poor fat camel with hump leaning at 90°, and (iv) meagre camel with empty hump placed on the side of the body. However, such classification is not sufficient to describe the full condition status of the animal.

Thus, the objectives of the present paper were (i) to describe the phenotypic variability within the Bactrian camel population in Kazakhstan based on their BM, (ii) to set up a grid and propose a body condition scoring based on the observation of hundreds of Bactrian camels with highly variable fat status.

## 2. Materials and Methods

### 2.1. Body Measurements

The body dimensions of Bactrian camels were measured by a standard meter ribbon. The following measurements were achieved between spring 2023 and autumn 2024 (i) length of the head from nose to occipital (HeadL); (ii) length of the neck (lower part) from base of head to the chest (NeckL); (iii) circumference of the neck at the middle of the neck (NeckC); (iv) height at the withers (HeightW); (v) girth circumference in front of the hump (ChestC); (vi) length of the front leg (LengthFL) from shoulder point to foot; (vii) length of the body (BodyL) from shoulder point to ischium point. As a whole, 477 Bactrian camels (from 7 months to 16 years old) were measured. They were split into 4 groups based on age, namely, young camel (YC) between 7 months and 2 years (n = 117), subadults (SA), 3–4 years old (n = 63), adults (AD) between 5 and 10 years (n = 201), and old adults (OA) more than 10 years old (n = 96). The animals belonged to 4 different camel farms from Kazakhstan in 3 very contrasting regions: one farm from Zhetisu region in the eastern part of the country (n = 207), one farm in Turkistan region in the southern part (n = 55), and two farms in Atyrau region in the north-west part, named Atyrau1 and Atyrau2 (n = 108 and 107, respectively). The maximal distance between the farms in Zhetisu and Ayrau region was around 3800 km.

### 2.2. Bactrian Camel Observation for BCS

Most of the observed Bactrian camels were in the same farms from Atyrau, Turkestan, and Zhetisu regions. In all farms, the farming system was under extensive management with mainly pastoral feeding. The assessment of the body condition of Bactrian camels was achieved in the frame of a monitoring of the Kazakh camel phenotypes, which included dromedaries (Aruana breed) and hybrids between Bactrian females (*Camelus bactrianus*) and dromedary (*Camelus dromedarius*) males [29]. To obtain a large variability of the body condition, the observations were taken at different seasons, as the access to natural resources could change throughout the year, and the potential feed supplementation was highly variable between farms and regions [30]. However, it was not possible to observe the animals in the wintertime due to the harsh climatic conditions and the difficulties in reaching the farms. In addition, a few photos of Bactrian with extreme BCS, originating from other countries, were considered to obtain a total panel of possible scores.

### 2.3. The Principle of Scoring

**Figure 1:**
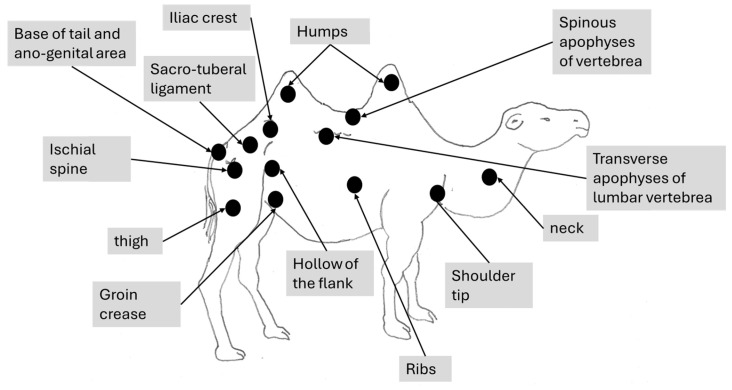
The main points to be observed for assessing BCS in the Bactrian camel.

The Bactrian camel was observed at a distance (2 to 3 m) based on visual criteria, using the assessment of the anatomical areas, usually taking into account the dromedary camel to assess the BCS [31]. It was not necessary to touch the animal, but sometimes, it could be helpful to confirm the visual judgment, especially when the fur of the Bactrian camel becomes abundant and leads to overestimation of the body condition. From back to front observations, the assessment included 12 main points (Figure 1):

1. The base of the tail and the ano-genital area.
2. The bones of the pelvis (iliac crest and ischial spine).
3. The sacro-tuberal ligament.
4. The thighs.
5. The transverse apophyses of the lumbar vertebrae.
6. The hollow of the flank.
7. The groin crease.
8. The spinous apophyses of the dorsal vertebrae.
9. The ribs.
10. The shoulder and, notably, the humerus joint.
11. The neck.
12. The state of repletion of the humps and the general appearance.

Usually, a BCS score is attributed to the profile (right side preferably) and the back of the animal, and an average of the two scores is provided, but can be modulated according to the general appearance. The assigned score could vary from 0 (very emaciated animal) to 5 (overweighed animal), and it is possible to score by ½ point.

To obtain a homogeneous scoring, only adult females were observed, notably because adult males have a different morphology and they are few in the herds (typically, one male for 30–40 females).

Then, a tentative scoring in 6 points (from 0 to 5) similar to that used for dromedary camels and bovines was proposed. One example of each score was taken in a photo from the side and from the back, and a drawing was completed to help the establishment of scoring. Then, a fine description of the different anatomical areas was clearly reported in a grid to define the different scores.

### 2.4. Statistical Analysis

Statistical analyses only concerned body measurement data, BCS being just a description. The objectives of the analysis were (1) to assess the age effect on the body measurements, and (2) to assess the regional effect on the adult animals, considering that their growth was stable. To achieve these objectives, the statistical procedure was as follows: (i) to apply a growth model (non-linear regression), several equations were tested: Grompertz, beta growth, exponential growth, logistic growth; (ii) to achieve a variance analysis (ANOVA) after homogenization of the variances to identify the parameters that are significantly different between farms (only for adults and old adults animals) and between class of age for all the animals; (ii) principal components analysis (PCA) of the body measurements parameters of the adult camels by using farms and age as supplementary variables, (iii) ascending hierarchical classification (AHC) to identify the homogeneous groups (clusters) of camels according to their body measurements and the relationships of these groups with the regions tested by Chi^2^.The convenient number of groups issued from the classification was based on the Hartigan index (adapted). The Hartigan index allows for assessing the quality of clustering solutions by quantifying the compactness of clusters and the separation between them. To compare our results with those of the literature data, a data table including the different Bactrian camel breeds in the world (as means of individuals) and some common body measurements (as variables) was built and analyzed by PCA and AHC. The references used came from China [7], India [8,31], Mongolia [32], and Russia [33].

The software used was XLstat-2024 (Addinsoft^©^, Paris, France).

## 3. Results

### 3.1. Body Dimensions of Bactrian Camels

Obviously, age is the main source of variation in the different body parameters, with increasing values from the group YC to OA. The relative changes in the dimensions of the different parameters were between 10.6 and 25% from the young group to the old adult group, with higher changes observed for neck length, chest circumference, and front leg length, the lowest being head length and body length (Table 1)

**Figure 2:**
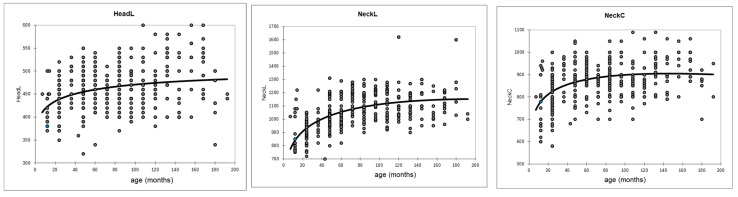

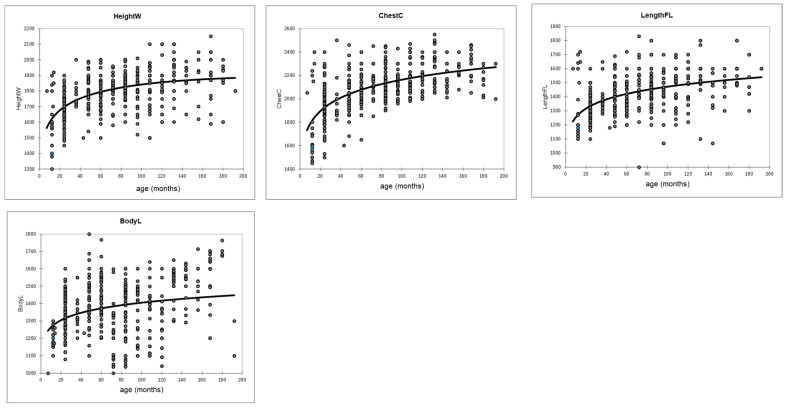
Growth curve of the body measurements in Bactrian camels according to the beta growth model.

Several growth equations were tested (Gompertz, beta growth, exponential growth, logistic growth). The most adjusted model with the minimum of residues was the beta growth equation: Y = pr1 × (1 + (pr2 − X1)/(pr2 − pr3)) × (X1/pr2)exp(pr2/(pr2 − pr3)) where pr1, pr2, and pr3 are the parameters of the model and X1 the age of the camel (in months). This model was applied for each measurement (Figure 2 and Table 2).

Except for headL and neck measurements (NeckL and NeckC), which were relatively stable after 48 months, all other measurements continued to increase, rapidly up to 4 years approximately, then more or less slowly. The growth of chestC appeared more continuous. As the data regarding the groups YC and SA were mainly collected in the farm from Zhetisu region (94% of the camels from YC group and 62% of the sub-adult group came from this region, only 6% and 28.5, respectively, were from Atyrau, while there were no measurements from the young group and only 9.5% were from sub-adult group in Turkestan), the regional effect was assessed based on the body measurements of adults and old adults only (Table 3).

**Figure 3:**
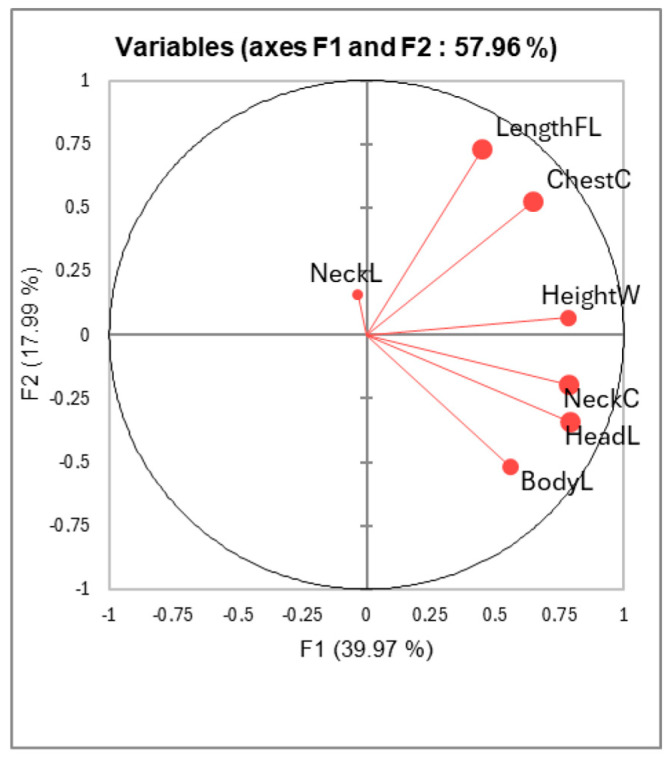
Correlations circle of the body measurements of the adult Bactrian camels on the two first factors of the PCA.

**Figure 4:**
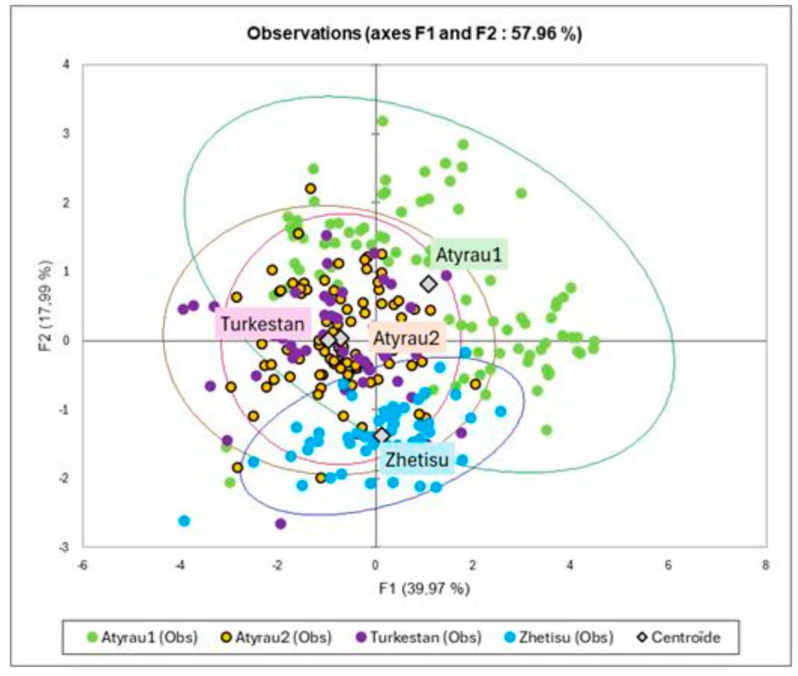
Main factorial plan (1, 2) of the PCA with the regions in supplementary variables showing the distribution of the observations colored according to the farm of origin, and their center of gravity of each farm (colored labels).

The correlations circle issued from PCA analysis has shown that all the parameters are correlated positively along the main factor, except LengthFL, which is correlated to the second factor, and NeckL, which is correlated to the third factor (Figure 3). The Bactrian camel from Atyrau1 had long legs, greater size, and a large chest, opposite to those from Zhetisu region, while there was a proximity between camels from Turkestan and Atyrau2, projected as close to the center of gravity of the main factorial plan (Figure 4). In these two farms, the Bactrian camels were characterized by their longer necks, while those from Zhetisu had longer heads, strong necks, and shorter legs.

**Figure 5:**
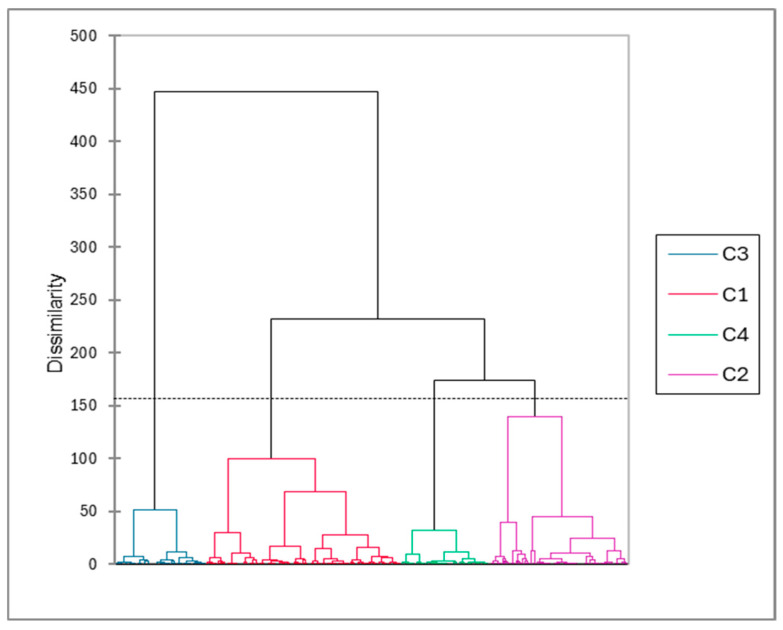
Dendrogram issued from the automatic classification (AHC) of the 278 adult Bactrian camels in Kazakhstan according to their body measurements. The four groups (namely C1 to C4) are represented by different colors.

The automatic classification confirmed the regional differences in body measurements: four main groups of animals (namely C1 to C4) can be identified by considering the optimal Hartigan index (minimum ratio within-cluster variance/between-cluster variance. Each group was described by the mean values of the measurements and the farm origin (Figure 5 and Table 4).

Thus, the Bactrian camels from Atyrau1were almost equally distributed in class1 and 3, corresponding to animals with long legs but differentiated by all other parameters, especially body length. The animals from Atyrau2 were more present in class2 (small head, long and fine neck, short leg, and medium values for the other parameters). A part (41.5%) was gathered in class1 (chi2 non-significant), characterized by a shorter head, long and fine neck, and very short body length and long legs. Camels from Turkestan were distributed similarly to the previous one, confirming the proximity of these two farms regarding the global conformation of their camels. In reverse, the camels from Zhetisu were mainly (84.5%) in class 4 and are characterized by short and fine necks, shorter height, chest circumference, and legs.

**Figure 6:**
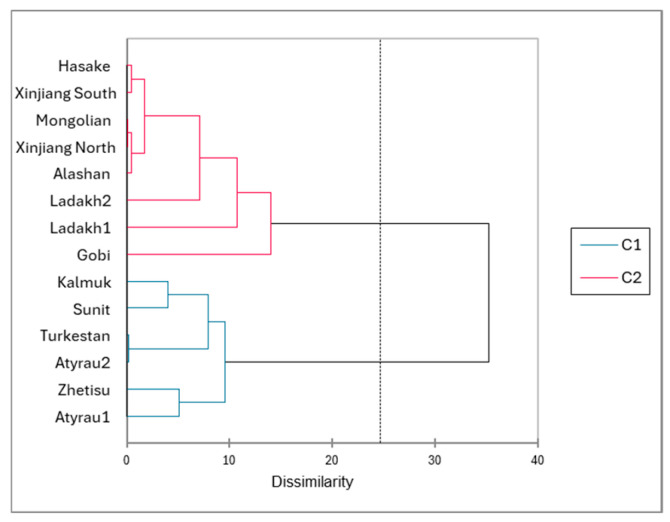
Dendrogram issued from the automatic classification (AHC) of the 10 groups of Bactrian camels reported in the literature, in addition to the present results.

The comparison with other published results was limited as few references were available: one reference in China, including six breeds, two references in India regarding the Ladakh breed, one Mongolian reference, and one Russian reference only. The automatic classification applied to the table of the mean values of body measurements gave a clear distinction between the Bactrian from Kazakhstan, Russia, and the Sunit breed from China on one side (C1 in Figure 6), and the other breeds from China, Mongolia, and India on the other side (C2 in Figure 6)

**Figure 7:**
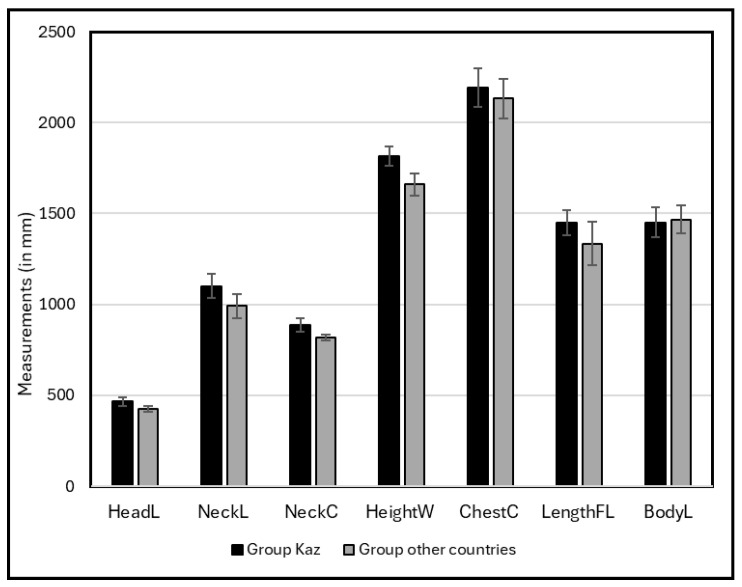
Values (mean and SD bars) of body measurements in Bactrian camels from Kazakhstan (group Kaz) and from other countries (group other countries).

Bactrian from the first group (Kazakh, Kalmuk, and Sunit breeds) had on average a bigger size for all parameters, except body length (Figure 7).

### 3.2. Body Condition Scoring

#### 3.2.1. Photos of “Examples” Corresponding to the Expected Scoring

**Figure 8:**
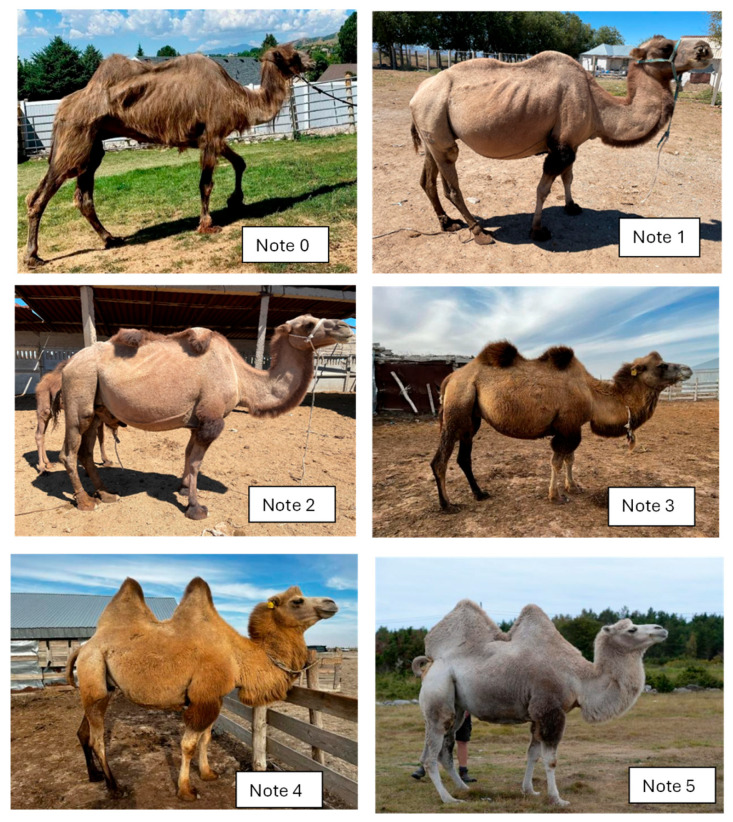
Examples of Bactrian camels corresponding to the expected BCS scoring.

The scoring was built by starting from 6 prototypes chosen among 56 photos and representing the six BCS notes from deeply meagre animals (note 0) to highly fat animals (note 5). The six photos are displayed below. Four of them were taken in Kazakhstan, the other two in Europe (Figure 8).

- Example note 0:

This photo was taken in France of a deeply parasitized camel coming from a circus. We can observe an important muscular melting with alopecia, lack of humps, deep hollowing of the flank, and an empty stomach. The animal has “the skin on the bones”.

- Example note 1

The camel was taken from a Kazakh farm in the Turkistan region. The ribs are still highly visible, and the humps are completely absent. The bones of the back are highly prominent, but compared to the former prototype, the hole of the flank is not well visible, and the belly is more rounded, probably because the camel had been watering recently.

- Example note 2

This camel came from the same farm as previously. If the ribs are still visible, the tuberosities of the basin are less visible, and the shoulder is more replenished. However, the two humps are small and still flexed, showing a very small fat reserve.

- Example note 3

It is a typical “medium” animal, coming from Kazakhstan. The ribs and the tuberosities, both in the shoulder and basin, are not visible, but the fat reserves in the humps are still limited, although the humps can be erected.

- Example note 4

This camel from Kazakhstan is in very good condition, with well-erected humps and well-covered basin, shoulder, and thorax. It is not easy to see any part of the skeleton.

- Example note 5

It is a very fatty camel from Sweden with its whole body widely covered in fat and very big humps, especially the rear one, with fat covering the basin. No parts of the skeleton are visible.

#### 3.2.2. Representation of the Scoring by Drawings

**Figure 9:**
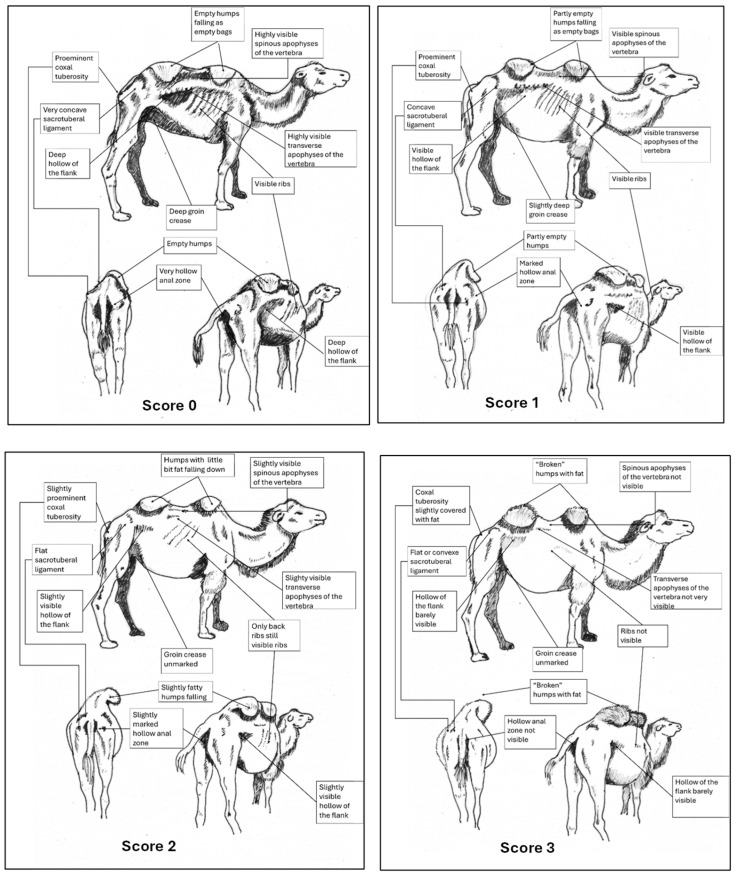

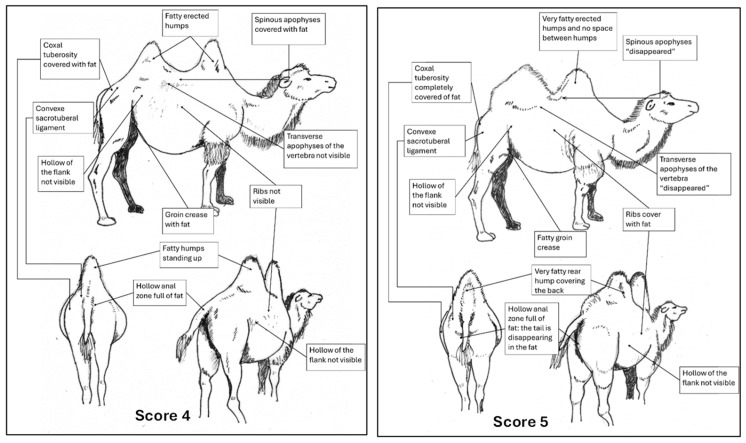
Drawings of the 6 levels of BCS scoring for Bactrian camels with the main comments.

From these six examples, it was possible to develop drawings from the side and back (Figure 9). Each drawing is commented on in order to propose a descriptive scoring proposed in the table below (Table 5).

#### 3.2.3. Description of the Scoring

The grid proposed in Table 5 is a detailed description of the scoring based on the field observations, partly adapted from the grid already established for dromedary camels [34].

## 4. Discussion

The differences in the body measurements observed in Kazakhstan could partly express breed diversity. Globally, three Kazakh Bactrian breeds were described in the country: *Oralobokeliki,* mainly present around the Caspian Sea and close to the Kalmuk breed from Russia, *Kyzylorda* in the central part of the country, and *Ontustik-Kazakhstan* in the south [35]. In the farms in Atyrau region, the breed *Oralobokeliki* is dominant, while in Turkestan, the *Ontustik* breed is more present. The majority of the phenotypes corresponding to group C3 and to a lesser extent to group C1 were from the Caspian Sea region (notably in the farm Atyrau1) and could correspond to *Oralobeliki* animals characterized by their huge height, long legs, and large chest (C3). However, if the camel herd appeared more homogeneous in this farm, a higher variability seems to be observed in the second farm (Atyrau 2) as well as in Turkestan region. In these two farms, the animals were equally present in cluster 1 (C1) and 2 (C2), i.e., corresponding to medium-sized animals which seem to correspond to *Kyzilorda* breed. The case of the farm in Zhetisu is specific: indeed, although it was in the eastern part of the country, the camel herd was formed with males from the Kyzylorda region and mixed with females from different regions. Unfortunately, there were few references regarding Bactrian camel body measurements, and the methodology used by the different authors was not necessarily homogeneous. Thus, the comparison with the data from the literature should be taken with caution.

BCS is regarded as a better indicator of fat storage than live weight, which depends on water and feed repletion, or on pregnancy stage. It is a useful tool, especially around parturition to detect the critical nutritional status of the camel, and to adjust the diet to meet the specific requirements when lactation is starting or for mating [23]. Its implementation is interesting at the herd level to detect feeding problems or management mistakes, but also at the animal level to detect, especially, parasitized camels or other health problems. Indeed, emaciation is an important consequence of faulty nutrient absorption and a symptom of many troubles, such as parasitism, specific deficiencies, or loss of appetite due to different metabolic or infectious diseases. In dairy cattle, for example, BCS was used in the prevention of metabolic diseases [36], to evaluate their relationships with reproduction performance [37], or with mastitis and other diseases [38]. BCS was also used in cattle and dromedary camels to detect trypanosomiasis, considering that animals with poor BCS were more susceptible to this disease, leading to the use of BCS in large-scale epidemiological studies [39]. In Nigeria, Woma et al. [40] investigated the relationships between BCS and the exposure of camels to the Peste des Petits Ruminants detected by serology. At reverse, very fatty animals (score 5) could be an indicator of an excess of feeds and a lack of optimization of resources, which can impact the farm economy.

BCS could also be fully integrated into welfare recommendations, as it can serve as an indicator of animal well-being, reflecting good management practices, including adequate feeding and proper health care, including for camels [41,42,43]. In the dromedary camel, BCS was also a parameter involved in the assessment of reproductive performance [44], such as ovarian activity [45] or reproductive abnormalities [46]. The relationships between BCS and the meat quality of the dromedary camel were also investigated [47].

Obviously, the proposed scoring needs to be validated to assess its reproducibility (ability of different assessors to assign the same score to a given animal) and repeatability (ability of each assessor to assign the same score several times for a given animal). The field application should require specific training of stakeholders (practitioners, technicians, scientists, farmers) in the case of monitoring, survey, or surveillance of camel herds. Such training is necessary for a better understanding of the assignment and to obtain a homogeneous point of view.

The application of BCS in the field would reduce the subjectivity of the stakeholders, and it is interesting to appreciate the homogeneity or heterogeneity of the body conditions within a herd, giving an appreciation of the farm management. For example, homogeneously high or low scores in a herd can mean a feeding problem (lack of energy in the ration in the first case, or excess nitrogen and energy in the second case) or a parasitosis affecting the entire herd. Conversely, a herd with high variability in BCS can mean the presence of an infectious or metabolic disease affecting a few animals or welfare problems, with some animals unable to access their feed ration. In the present paper, three supports were used: the selection of photos as examples of expected scoring, a unique representation of each score by drawings, and a detailed grid. The photos were an illustration of different “archetypes” for a specific score, but some differences could occur between breeds or ecotypes, and generally, the choice of drawing is regarded as a better representation and provides uniformity in the assessment of the different anatomical landmarks [21]. Drawings were widely used for the establishment of BCS in other species such as Holstein cows [48] and different tropical species as zebu, sheep, goat, or donkey [21]. The grid is especially useful for training and is regarded as a complementary tool for drawings. This grid can be distributed with the drawings in brochures adapted for breeders, as it was created for the dromedary in the reference cited above [34].

**Figure 10:**
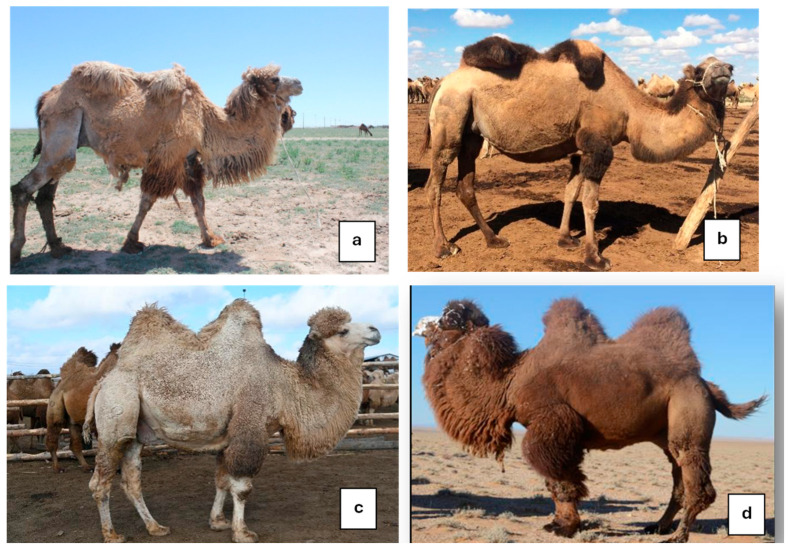
The seasonal change of Bactrian camel’s fleece: (**a**) in Spring (molting period), (**b**) in summer, (**c**) in autumn, (**d**) in winter.

However, the assessment of BCS for Bactrian camels faces different pitfalls: (i) the seasonal abundance of wool; (ii) the behavior of the humps; (iii) the importance of hybridization. Contrary to the dromedary camel, the Bactrian camel has abundant fur, especially in winter. In spring, the animal molts, losing its fleece, which is generally collected by combing, and the wool gradually grows back until the following winter. In such conditions, scoring is difficult in the wintertime, and not only because of the climatic conditions (Figure 10).

Consequently, it is preferable to assess the BCS after molting or shearing, up to the middle of autumn, especially since palpation is difficult unless it is possible to approach the animals in a milking corridor when it exists (and only lactating females). Usually, palpation is an easy observation for small ruminants such as sheep and goat, but it requires specific logistic conditions for big animals such as Bactrian camels, which are sometimes not easy to approach, especially dried females, which have spent the season in the steppe.

As said above, Bactrian humps can be “broken” despite their replenishment with fat, and this can be an individual specificity. Moreover, when the hump is flexed like a more or less empty bag, it could be on one side or another, each hump independently. In addition, for example, in Mongol tradition, there is a specific name according to the side and the shape of the humps, as well as their movements during walking (Table 6).

Since the “filling” of the humps with fatty tissue may appear different for the front one and the back one, the final score should be adjusted to take these variations into account. For example, in the case of one hump being well erected and one appearing empty, the score can be reduced by half a point. In dromedaries, the estimation of the hump volume and weight was proposed by considering it as a semi-ellipsoid measurable with the formula V = ½ (4/3 π × rL × rW × rH) where rL, rW, and rH are, respectively, the radius of length, width, and height of the hump, the weight being estimated from charts obtained on slaughtered animals by the formula √P(kg) = 1.59 + 0.0836 H (cm) [50,51]. However, in Bactrians, hump asymmetry is common, especially on very fat animals, leading to an underestimation of the hump fat reserve. This limit of the method to assess the hump fat reserve is accentuated in hybrids.

**Figure 11:**
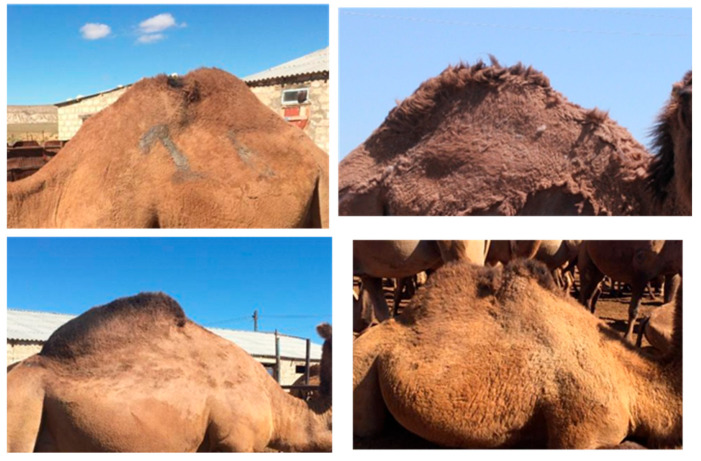
Different forms of the hybrid humps at different levels of hybridization.

Indeed, if in China and Mongolia, only Bactrian camels are reared [52,53], in central Asia, the hybridization between dromedary and Bactrian camel is common [54], especially in Kazakhstan [55]. The fat reserve in the humps of the hybrids becomes more difficult to assess because a high variety in hump shape can be observed (Figure 11).

The BCS proposed for Bactrian could be used for hybrids, but with the necessary adaptation to the hybridization level, with the two humps being completely fused or more or less separated according to the type of crossbreeding (father Bactrian or dromedary) and generation (F1, F2, F3).

In Kazakhstan, a state-regulated grading system is applied to all farm animals to establish conformity with recognized pure breed standards. For camels, grading assessments are conducted at 2.5 and 5 years of age. The evaluation is based on a set of parameters encompassing phenotypic traits, zootechnical characteristics, and selected genetic indicators. Due to the complexity of body condition scoring (BCS), it could serve as a valuable additional criterion to be incorporated into future revisions of camel grading standards.

In the future, it could be useful to use a 3D imaging device, although remarkable progress has been made. This technology was tested on dairy cows with interesting results, allowing for measuring morphological parameters, volume of body, and modeling of the complete shape of the animal [56,57]. However, the scoring of body condition could contribute to a better interpretation of such a model. Moreover, such a device could be more useful in a context of research than in field conditions.

In camels, both in dromedary and in Bactrian, some studies were focused on the use of digital technology, notably the 3D-modeling method, compared to manual body measurements and the photographing method, to determine the morphological features of camels [58]. However, even if this methodology is easy and practical, it is useful to obtain accurate body measurements and eventually to estimate the weight of the animal [59], but not yet to evaluate the body condition score.

## 5. Conclusions

The significant relationships between homogeneous groups of Bactrian camel and their region of origin support the idea of their belonging to a specific breed. However, a better standardization of body measurements should be proposed to obtain a clear idea of the diversity in Bactrian camel populations, leading to easier geographical comparison. Elsewhere, the use of a BCS as a tool for assessing the feeding management or the health status of the Bactrian camel can be developed easily, but it requires training by regular practical sessions to have experienced technicians and veterinarians, able to transmit their know-how. It is also useful for scientists to assess the relationships between BCS and different production and health performances, or the links with the feeding practices and availability of resources. It is expected that the present scoring developed for the first time for an emblematic species of central Asia will usefully complement the “toolbox” of producers, veterinarians, and other stakeholders in Bactrian breeding to assess its good management.

## Figures and Tables

**Table 1:** Mean body measurements (in mm) of Bactrian camels in Kazakhstan at different ages: young camel (YC), sub-adult (SA), adult (AD), old adult (OA); n = 477.

Agegroup	HeadL	NeckL	NeckC	HeightW	ChestC	LengthFL	BodyL
OA	481.3 ^c^	1136.9 ^d^	909.1 ^c^	1877.9 ^c^	2235.7 ^d^	1514.4 ^d^	1463.3 ^c^
AD	463.4 ^b^	1095.8 ^c^	884.4 ^b^	1813.2 ^b^	2121.3 ^c^	1449.5 ^c^	1356.6 ^b^
SA	459.3 ^b^	1018.7 ^b^	882.1 ^b^	1785.5 ^b^	2016.8 ^b^	1406.7 ^b^	1395.7 ^b^
YC	435.2 ^a^	909.8 ^a^	810.7 ^a^	1680.3 ^a^	1908.4 ^a^	1316.2 ^a^	1318.5 ^a^
Total	459.6 ± 46.1	1048.3 ± 139.8	871.0 ± 85.1	1790.0 ± 131.2	2078.3 ± 195.4	1424.2 ± 145.7	1373.9 ± 156.1
% Increase	10.6	25.0	12.1	11.8	17.2	15.1	11.0
CV	0.100	0.133	0.098	0.073	0.094	0.102	0.114
*p* value	*<0.0001*	*<0.0001*	*<0.0001*	*<0.0001*	*<0.0001*	*<0.0001*	*<0.0001*

^a,b,c,d^ The values with different subscripts in the column differ significantly; CV = coefficient of variation (HeadL): length of the head; (NeckL) length of the neck; (NeckC) circumference of the neck; (HeightW) height at the withers; (ChestC) girth circumference; (LengthFL) length of the front leg; (BodyL) length of the body.

**Table 2:** Beta growth equations according to age for the different body measurements in Bactrian camels from Kazakhstan (ages between 7 months and 16 years).

Parameter	Beta Growth Equation
HeadL	HeadL = 488.7 × (1 + (399 − age)/7040) × (age/399)^0.056^
NeckL	NeckL = 1153.6 × (1 + (203 − age)/1265) × (age/203)^0.16^
NeckC	NeckC = 904 × (1 + (146 − age)/1568) × (age/146)^0.093^
HeightW	HeightW = 1892 × (1 + (271 − age)/3856 × (age/271)^0.070^
ChestC	ChestC = 2430.5 × (1 + (908 − age)/10,498) × (age/908)^0.086^
LengthFL	LengthFL = 1871 × (1 + (7861 − age)/112.262) × (age/7861)^0.070^
BodyL	BodyL = 1709 × (1 + (17.451 − age)/374.834) × (age/17.451)^0.046^

**Table 3:** Mean body measurements (in mm) of Bactrian camels from different regions in Kazakhstan (n = 298).

Region	HeadL	NeckL	NeckC	HeightW	ChestC	LengthFL	BodyL
Zhetisu	494.9 ^b^	1009.3 ^a^	939.0 ^c^	1816.2 ^a^	2025.9 ^a^	1394.0 ^a^	1456.9 ^b^
Atyrau1	490.4 ^b^	1057.3 ^b^	914.6 ^b^	1894.9 ^b^	2282.4 ^c^	1581.5 ^b^	1354.0 ^a^
Atyrau2	448.0 ^a^	1189.0 ^c^	860.5 ^a^	1810.0 ^a^	2132.0 ^b^	1426.6 ^a^	1384.3 ^a^
Turkestan	438.0 ^a^	1175.5 ^c^	854.7 ^a^	1782.4 ^a^	2122.4 ^b^	1427.8 ^a^	1399.0 ^a,b^
Total	469.2 ± 46.6	1109.1 ± 116.5	892.4 ± 71.4	1834.1 ± 115.9	2158.3 ± 145	1470.5 ± 134.8	1391.1 ± 169.1
CV	0.099	0.105	0.080	0.063	0.067	0.092	0.122
*p* value	*<0.0001*	*<0.0001*	*<0.0001*	*<0.0001*	*<0.0001*	*<0.0001*	*0.003*

^a,b,c^ The values with different subscripts in the column differ significantly; CV = coefficient of variation.

**Table 4:** Mean values of the body measurements of the 278 adult Bactrian camels in the four groups (namely C1 to C4) issued from the automatic classification (tested by ANOVA) and distribution of the farms in the different groups (the values in bold tested by chi^2^ test were significantly higher). The colors of the headings are corresponding to the colors of the different clusters presented in Figure 5 above.

Class Parameter	Class3 n = 53	Class1 n = 113	Class4 n = 52	Class2 n = 79	*p* Value
HeadL	533.4 ^c^	441.6 ^a^	455.0 ^b^	449.2 ^a,b^	*<0.0001*
NeckL	1055.5 ^b^	1120.4 ^c^	962.4 ^a^	1192.7 ^d^	*<0.0001*
NeckC	971.9 ^c^	868.4 ^b^	858.4 ^a,b^	843.8 ^a^	*<0.0001*
HeightW	1965.1 ^d^	1783.8 ^b^	1739.4 ^a^	1829.7 ^c^	*<0.0001*
ChestC	2340.8 ^d^	2180.6 ^c^	1958.8 ^a^	2107.1 ^b^	*<0.0001*
LengthFL	1519.6 ^b^	1551.9 ^b^	1359.8 ^a^	1366.8 ^a^	*<0.0001*
BodyL	1535.9 ^d^	1261.1 ^a^	1368.3 ^b^	1443.0 ^c^	*<0.0001*
Atyrau1	**45.8%**	**52.1%**	0%	2.1%	*<0.0001*
Atyrau2	1.1%	41.5%	3.2%	**54.3%**	*<0.0001*
Turkestan	2.0%	**49.0%**	0%	**49.0%**	*<0.0001*
Zhetisu	12.1%	0%	**84.5%**	3.45%	*<0.0001*

^a,b,c,d^ The values with different subscripts in lines differ significantly.

**Table 5:** Grid of the body condition scoring for Bactrian camels.

	Back					Flanc				
Scores	Ano-Genital Region	Ischial Spine	Iliac Crest	Sacrotuberal Ligament	Thigh	Hollow of the Flank	Transverse Apophyses	Spinous Apophyses	Crease of Groin	Ribs
**0**	Very deep at the base of the tail	Very prominent	Completely visible	Very concave	Deeply concave	Very deep	All prominent	All visible even under the humps	Deep crease of groin completely dry	All visibles (“skin on bones”)
**1**	Deep, base of the tail still prominent	Well visible	Still visible	Concave	Still oncave	Clearly apparent	Still prominent	Still visble on the back	Slightly deep and dry crease	Clearly visible
**2**	Visible hollow	Well visible	Slighly visible	Flat	Flat	Visible	Slightly visible	Slightly visible on the back	Unmarket crease	Back ribs slighly visibles
**3**	Slight hollow	Slightly visible, cover of fat	Slightly covered of fat	Flat to convex	Slightly convex	Very slight	Not very visible	Not very visible	Unmarked crease but very small quantity of fat	Invisible
**4**	Filled	Hardly visible and covered of fat	Covered of fat	Convex	Convex	Almost invisible	Invisible	Well covered by fat	Fatty crease	Covered of fat
**5**	The base of tail is covered of fat	Disappeared in fat	Disappeared in fat	Stronglt conex	Well rounded	Totally invisible	Invisble and rounded back	Rounded back	Crease compltely covered of fat (rounded surface)	Presence of intercostal fat

**Table 6:** The different positions and movements of the Bactrian camel humps and their names in the Mongol tradition [49].

Hump Shape	Description	Denomination in Mongol	Phonetic Translation
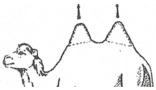	Two humps well erected	*Tyг шиpээ*	*Tug chyréé*
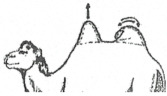	Raised front hump Rear hump flexed on the right side	*coëo*	*Soyo*
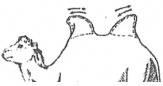	Front hump flexed forward and rear hump flexed backward	*Coлбин шиpээ*	*Solbyn chiréé*
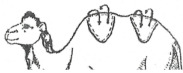	The two empty humps flexed on the left side	*Зэв Бypyy лөг*	*Zev Bourou leg*
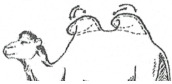	The two humps flexed on the right side	*Xaнaн*	*Khanan*
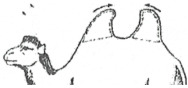	Front hump flexed backward and rear hump flexed forward	*Дoтoгyйoo чaчиp*	*Dotoguyoo chachyr*
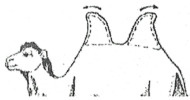	Front hump flexed forward and rear hump flexed backward	*Гaдaгчaa чaчиp*	*Gadagchaa chachyr*
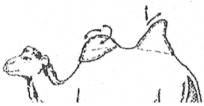	Front empty hump flexed forward and asymmetric erected rear hump	*Xoйт бox чaчиp*	*Khoyt bokh chachyr*
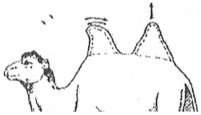	Font hump moving forward and symmetric erected rear hump	*Уpд бox чaчиp, xoйт чиpээ*	*Ourd bokh chachyr, khoyt chiréé*
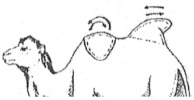	Front empty hump flexed on the right side and rear erected hump, flexed backward	*Уpд бөx лөг, xoйт бөx Xaнaн*	*Ourd bokh leg, khoyt bokh khanan*
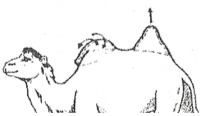	Front empty hump flexed on the right side and well erected rear hump	*Coлбын coëo чиpээ*	*Solbyn soyo chiréé*
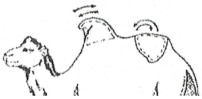	Front erected hump flexed forward and empty rear hump flexed on the left side	*Уpд бөx coлбын, xoйт бөx лөг*	*Ourd bokh solbyn, khoyut bokh leg*

## Data Availability

All data generated or analyzed during this study are included in this published article.

## Abbreviations

The following abbreviations are used in this manuscript:

AD	Adult
AHC	Ascending Hierarchical Classification
BCS	Body Condition score
BM	Body measurement
OA	Old adult
PCA	Principal Components Analysis
SA	Subadult
YC	Young Camel
